# Pharmaceutical interventions for drug-related problems in the neonatal intensive care unit: incidence, types, and acceptability

**DOI:** 10.3389/fphar.2024.1391657

**Published:** 2024-05-30

**Authors:** Norhan Attia Ahmed, Ehab Ahmed Fouad, Osama M. El-Asheer, A. S. M. Ghanem

**Affiliations:** ^1^ Department of Clinical Pharmacy, Faculty of Pharmacy, Assiut University, Assiut, Egypt; ^2^ Department of Pharmaceutics, Faculty of Pharmacy, Assiut University, Assiut, Egypt; ^3^ Department of Pediatrics, Faculty of Medicine, Assiut University Hospital, Assiut, Egypt

**Keywords:** pharmaceutical care network of europe classification (PCNE), drug-related problems (DRPs), neonatal intensive care unit (NICU), clinical pharmacy services, clinical pharmacist interventions, Egypt

## Abstract

**Background:** Drug-related problems (DRPs) are widespread in hospitalized neonates, but studies on the prevalence of DRPs in this population are limited. The presence of clinical pharmacists on multidisciplinary teams helps prevent and reduce DRPs.

**Aim:** This investigation aimed to identify and classify the incidence of DRPs in the neonatal intensive care unit (NICU), to determine the determining factors associated with DRPs and to document clinical pharmacists’ interventions, outcomes, acceptance rates and clinical significance.

**Method:** A prospective descriptive hospital study was conducted from August to November 2023 at the NICU of Children’s University Hospital, Assiut University, Egypt. DRPs were classified using the Pharmaceutical Care Network of Europe (PCNE) classification V9.1.

**Results:** Three hundred sixteen neonates were included in the study, with a mean gestational age of 34 ± 4 weeks and a mean birth weight of 2.03 ± 0.85 kg. A total of 1723 DRPs occurred among 283 neonates (89.6%), an average of 5.5 ± 5.1 DRPs per patient. The main types were treatment effectiveness (P1) (799, 46.4%), followed by others (P3) (469, 27.2%), and treatment safety (P2) (455, 26.4%). The leading causes were dose selection (C3) (1264, 61.9%) and “other domain” (C9) (543, 26.6%). Of the 2149 interventions introduced by pharmacists, 98.8% were accepted and 93% were accepted, and fully implemented. As a result, 92% of the DRPs were resolved. Both length of hospital stay and number of medications were significantly associated with DRPs.

**Conclusion:** DRPs are common in the NICU; this study demonstrated the crucial role of clinical pharmacists in identifying and resolving DRPs.

## 1 Introduction

Drug-related problems (DRPs) are a significant concern in the healthcare sector, with over half of the harm to patients being preventable (Panagioti et al., 2019; Hodkinson et al., 2020). According to the Pharmaceutical Care Network of Europe (PCNE) classification, a drug-related problem (DRP) has been defined as “an event or circumstance involving drug therapy that actually or potentially interferes with desired health outcomes” (Pharmaceutical Care Network Europe Association, 2020.).

The risk of DRPs is exceptionally high in critically ill ICU patients, particularly neonatal infants, due to their weight varying with age and metabolic differences between adults and children. Most drugs used for newborns are either unlicensed or off-label, which can lead to dosage errors (Sorrentino and Alegiani, 2012). The few published studies conducted in the neonatal intensive care unit (NICU) reported that DRPs were common in the NICU, with an incidence ranging from 41.7% to 82% (Leopoldino et al., 2019a; 2019b; Nascimento et al., 2020; Awoke et al., 2021). Additionally, one study reported that the incidence of DRPs was more significant in the NICU than in the pediatric intensive care unit (Rashed et al., 2014).

The roles and responsibilities of pharmacists differ across countries; in the United Kindom, South Africa, the United States, and Australia, pharmacists mainly participate in ward rounds, medication chart reviews, therapeutic drug monitoring (TDM), and providing information to healthcare (Krzyzaniak and Bajorek, 2017). In Europe (France, Quebec, Switzerland, and Belgium), clinical activities incorporate chart review, clinical research, medication distribution, staff education, and addressing inquiries related to medication information. (Prot-Labarthe et al., 2013).

A cross-sectional survey was performed to explore Australian and Polish pharmacy practices. Australian clinical pharmacists were more integrated into the NICU team, focusing on medication review and actively intervening to address drug-related problems and provide information to the healthcare. In contrast, Polish pharmacy practices focused more on dispensing-based roles and medication supply (Krzyzaniak et al., 2018). This study demonstrated the lack of standardization in services across countries, highlighting the importance of standardized care for critically ill patients. (Krzyzaniak and Bajorek, 2017).

Numerous studies have explored the role of clinical pharmacists in NICUs, including assessments of the acceptability of pharmacist interventions (Khan et al., 2016; Leopoldino et al., 2019a; Nascimento et al., 2020; Yalçın et al., 2023).

In Egypt, the clinical pharmacy specialty was introduced in 2006, necessitating efforts to standardize and document clinical pharmaceutical practices (Abdel-Latif and Sabra, 2016; Ali et al., 2018). No previous studies have explored the incidence of DRPs in NICUs and associated pharmaceutical interventions. Only one study has examined DRPs among pediatric patients in the medical wards of a tertiary cardiac care center in Cairo, demonstrating a high incidence of DRPs (Sabry et al., 2014). Consequently, there is a need to assess and document clinical pharmacy activities in NICUs to facilitate expanding clinical pharmacy services.

This study aimed to ascertain the incidence, types, and causes of DRPs; assess the preventability of DRPs; identify associated risk factors and the most commonly involved medications; estimate the frequency and nature of clinical pharmacists’ interventions; evaluate the acceptability, and outcomes of these interventions; and determine their significance.

## 2 Methods

### 2.1 Study design and setting

A prospective descriptive hospital study was conducted at the 42-bed NICU of Children’s University Hospital, Assiut University, a tertiary teaching hospital, from August to November 2023.

Clinical pharmacists work from Saturday to Friday (8:00 a.m.–2:00 p.m.) and are present in the unit even during official holidays, ensuring continuous patient care and fulfilling their responsibilities. The NICU employs twelve clinical pharmacists, all licensed by the Egyptian General Administration of Licensing and Commissioning, each with at least 2 years of experience. The unit provides services for babies at Women’s Health Hospital-Assiut University and out-born babies.

Pharmacists attend the ward rounds with the junior and senior physicians. Any identified DRPs are discussed with the physicians and documented using the standardized intervention form.

### 2.2 Participants (inclusion and exclusion criteria)

All neonates admitted during the study period were included as long as their stay exceeded 24 h and they received at least one drug; otherwise, they were excluded.

Medications excluded from this study comprised electrolyte solution, total parenteral nutrition, whole blood, and diagnostic agents, as they were not categorized as drugs.

Patients were followed from admission until.

• Discharge
• Transfer
• Death

### 2.3 Operational definition

A drug-related problem is “an event or circumstance involving drug therapy that actually or potentially interferes with desired health outcomes” (Pharmaceutical Care Network Europe Association, 2020).

### 2.4 Data collection

One of the investigators (authors) was responsible for collecting data from the hospital daily. Data were collected from the patient’s medical charts, physician notes, nurse records, and institutional clinical pharmacy unit records.

For each neonate, the following data were collected: age, sex, birth weight, route of delivery (vaginal delivery or cesarean delivery), presence of premature rupture of membranes, diagnosis, and length of hospital stay.

In addition, the number of medications and the number of clinical conditions were collected to assess the determinants of DRPs.

### 2.5 DRP identification and assessment

**FIGURE 1:**
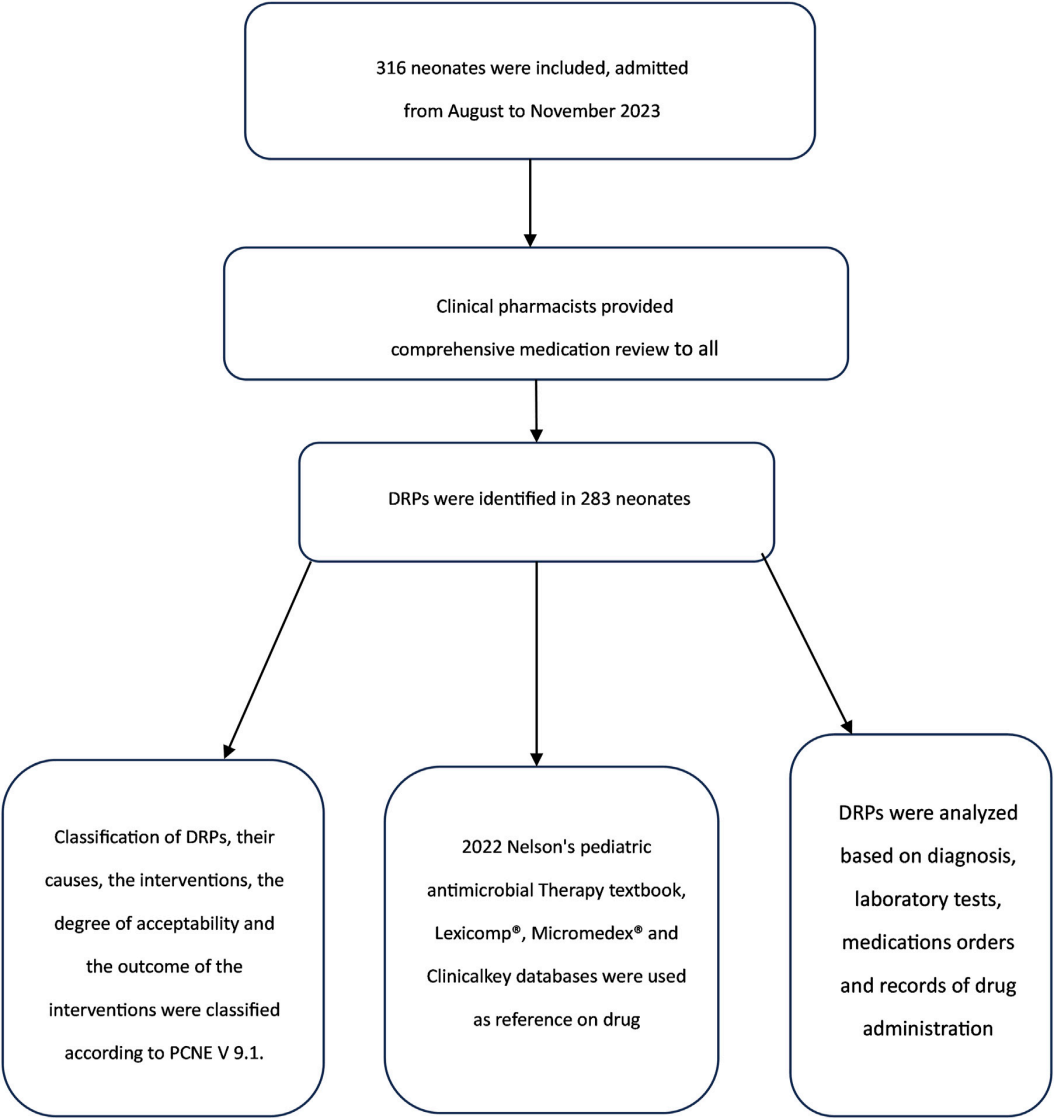
Flowchart describes the process of identification of DRPs.

Figure 1 depicts a flowchart illustrating the processes and tools for identifying DRPs.

DRPs, their causes, interventions, degree of acceptability, and outcomes were classified according to PCNE V 9.1.

The PCNE classification consists of primary domains for problems, causes, interventions, level of acceptance of these interventions and problem status. Each domain has its subdomains. Problems (P) are divided into three domains: “treatment effectiveness (P1),” addressing potential issues with pharmacotherapy effectiveness, “treatment safety (P2),” focusing on adverse drug events (ADE), and “Other (P3),” including “unnecessary treatment (P3.1)” and “unclear problem (P3.2).” In this study, the unclear problem (P3.2) was considered for any problem not included in the other domains and subdomains, like incomplete prescription.

For causes (C) of DRPs, there are nine domains, including “other (C9),” which encompasses “inappropriate outcomes monitoring (C9.1),” “other cause (C9.2)” and “no obvious cause (C9.3).” The “other subdomain (C9.2)” was utilized for causes not covered in the other subdomains, like incomplete prescriptions. Interventions (I) are divided into four domains. Drug level interventions (I3) encompass various actions such as “drug changed to” (I3.1), “dosage changed to” (I3.2), “formulation changed to” (I3.3), “instructions for use changed to” (I3.4), “drug paused or stopped” (I3.5) and “drug started” (I3.6) Pharmacist interventions, including dose calculations for incomplete prescriptions, are categorized under the “other intervention (I4.1).”(Pharmaceutical Care Network Europe Drug Related Problem Classification, 2020).

Since patients were not part of the study, patient-level interventions were not included in the intervention category.

Interventions proposed to or discussed with physicians related to drug changes were classified at the drug level (I3) to ensure comprehensive documentation.

### 2.6 DRP preventability and clinical significance of interventions

The degree of DRPs’ preventability and the interventions’ clinical significance were evaluated by a panel consisting of academic clinical pharmacists (authors) and a senior physician (author). For each event, the panel discussed the clinical significance of interventions and degree of preventability of DRPs until reaching a consensus.

The degree of preventability of DRPs was assessed using the modified Schumock and Thorton’s scale (Schumock and Thornton, 1992), consisting of three sections: Section A: “Definitely preventable” DRPs include instances of previous drug reactions, inappropriate drug selection for the patient’s clinical condition and incorrect dosing for the patient’s age, weight, or disease. Section B: “Probably preventable” DRPs encompass situations where TDM or laboratory tests were not conducted, documented drug interactions occurred, or preventative measures were inadequate. Section C: “ Not preventable” DRPs are those who deemed unavoidable.

The clinical significance of the interventions was evaluated using a French scale adapted from Hatoum et al. (Hatoum et al., 1988).

Which comprises four levels: (Chedru and Juste, 1997):

Level 0: No impact on the patient because the intervention has no consequences or is performed after the event (informational)

Level 1: Significant impact enhancing treatment efficacy, patient safety and quality of life (standard of practice).

Level 2: Very significant impact, preventing organ dysfunction or irreversible sequel.

Level 3: Vital impact, averting a fatal accident.

Furthermore, the study aimed to determine the most common drugs involved in DRPs, so all prescribed medications during the hospital stay were collected.

Pharmaceutical and medical terminologies were standardized using the Anatomical Therapeutic Chemical (ATC) classification for drugs (WHO-ATC) (WHO Anatomic Therapeutic Chemical Classification, 2023b) and the online version of the International Classification of Diseases version 11 (WHO-ICD 11) for disease categorization (International Classification of Diseases Version 11, 2023).

Incidence of DRPs

$$\text{Incidence of DRPs}=\frac{Number\;of\;patients\;with\;at\;least\;one\;DRP}{Total\;number\;of\;patients\;included\;in\;the\;study}\times 100$$

For each drug**, the risk ratio** was calculated as follows: $\frac{number\;of\;times\;the\;drug\;was\;involved\;in\;DRP}{number\;of\;times\;the\;drug\;was\;prescribed}$

### 2.7 Statistical analysis

The data were input and coded into Microsoft Excel 365 and subsequently exported to Statistical Package for Social Sciences (SPSS) version 26 for statistical analysis.

Descriptive statistics were used to measure variables related to patient demographics, types of DRPs, and drugs associated with different DRPs.

Categorical data were expressed as frequency and percentage, while continuous data were expressed as mean ± SD and median (interquartile range, IQR), as appropriate.

The chi-square test was employed to identify whether DRP and mortality were related.

In the present study, univariate and multivariate logistic regression analyses were performed using SPSS V.26 to identify the factors related to DRPs.

Variables included: gender, age, birth weight, gestational age (less than 37 weeks), mode of delivery, premature rupture of membranes (PROM), number of medications, number of clinical conditions, disease category, and length of hospital stay.

The multivariate model included only variables significant at *p* ≤ 0.05 in the univariate analysis.

Crude odds ratio (COR) and adjusted odds ratio (AOR) with 95% confidence intervals (CI) were used to report the findings of univariate and multivariate analyses, respectively. A *p*-value <0.05 was considered to indicate statistical significance.

### 2.8 Ethics approval

Approval number 05-2023-003 was obtained on 2 August 2023, from the “Research Ethics Committee” of the Faculty of Pharmacy, Assiut University, Egypt.

## 3 Results

### 3.1 Patient enrollment in the study

During the study period, 373 neonates were admitted to the NICU. Among them, 57 neonates were excluded: 14 patients had no medication and 43 had a duration of stay less than 24 h. Finally, 316 neonates were included in this study.

### 3.2 Demographics and clinical statistics

The study population consisted of 55.1% male patients (174) with a mean gestational age of 34 ± 4 weeks and a mean birth weight of 2.03 ±0. 85 kg. The neonates were hospitalized for a median of 12 days (range: 2–81 days). The mortality rate was 33% (104), as shown in Table 1, and there was no association between DRPs and the mortality rate (*X* ^2^ = 0.199, df = 1, *p* = 0.656).

**TABLE 1:** Demographic and clinical characteristics of the study population (*n* = 316).

Characteristics	Value
**Gestational age (Weeks) (mean** ± **SD)**	34 ± 4
*Gender*	
** Male (n, %)**	174 (55.1)
**Birth Weight(kg) (mean** ± **SD)**	2.03 ± 0. 85
**Length of hospital stay (days) (median, IQR)**	12 (2–81) days
**Age at admission (hours^a^, days) (median, IQR)**	Hours (hours-27 days)
*Route of delivery*	
** Vaginal delivery (n, %)**	55 (17.4)
**Premature Rupture of membrane (PROM) (*n*, %)**	45 (14.2)
*Disease category* (Top three)^b^	
** Infection (*n*, %)**	184 (58.2)
** Respiratory disorder (n, %)**	163 (51.6)
** Congenital anomalies (n, %)**	115 (36.4)
**Number of medications used (mean** ± **SD)**	5.9 ± 4.3
**Deaths (n, %)**	104 (33)

***IQR*, interquartile range**.

^a^ **Hours, age less than 24 h**.

^b^ Diseases were classified according to WHO-ICD, 11, More than one disease was identified in some cases.

**Percentage of disease type was calculated based on number of patients with specific disease divided by total number of patients multiplied by 100%**.

### 3.3 Incidence of DRPs

A total of 1723 DRPs occurred in 283 patients, with a mean of 5.5 ± 5.1 DRPs per patient. The incidence of DRPs was 89.6% (Table 2). There were 33 (10.4%) patients with no DRPs, 32 (10.1%) with one DRP, 43 (13.6%) with two DRPs and 208 (80.2%) with three or more DRPs.

**TABLE 2:** Frequency and incidence of drug-related problems in the NICU.

Characteristics	n, %
**Number of DRPs**	1723
**Number of patients with DRPs**	283 (89.6)
*Number of patients with DRPs stratified by gender*	
**Male**	154 (54.4)
**Female**	128 (45.2)
**Unknown**	1 (0.4)
*Number of patients with DRPs stratified by type of DRPs ^a^*	
**Treatment effectiveness**	244 (86.2)
**Treatment safety**	147 (51.9)
**Other**	81 (28.6)

^a^ Patients with different types of DRPs, do not add up to the total number of patients with DRPs, because a patient may have more than one problem. Instead, the percentage is determined by dividing the number of patients with a particular type of DRPs, divided by the total number of patients with DRPs.

### 3.4 Types and causes of DRPs

**FIGURE 2:**
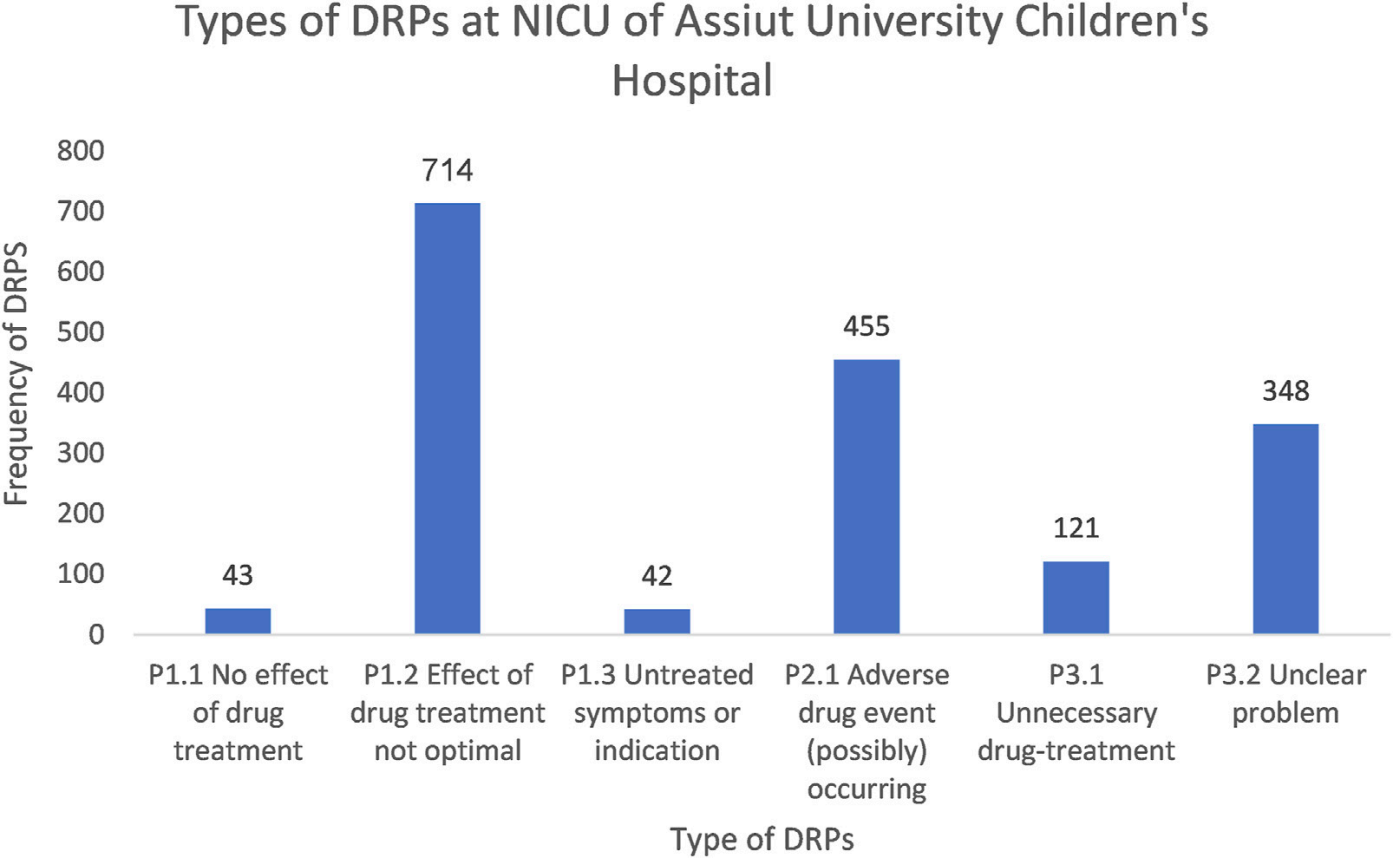
A chart showing the types of DRPs at the NICU of Assiut University Children’s Hospital based on PCNE classification V9.1.

The treatment effectiveness (P1) domain exhibited the highest frequency among the DRPs in the NICU, comprising 799 cases (46.4%). Specifically, the main issue observed was drug treatment effects not optimal (P1.2), accounting for 714 (41.4%), followed by others (P3) constituting 469 (27.2%), mainly unclear problems (P3.2), accounting for 348 (20.2%) and the last type treatment safety (P2) domain, mostly ADEs (possibly) occurring (P2.1), responsible for 455 (26.4%) (Figure 2).

A total of 2041 causes were identified for 1723 DRPS. The leading cause was in the dose selection domain, totaling 1,264 (61.9%). Specifically, the most prevalent issue within this domain was the administration of a dose that was too low, responsible for 563 cases (27.6%), followed by “other domain” accounting for 543 cases (26.6%), and mainly other causes accountable for 541 (26.5%) (Table 3).

**TABLE 3:** Causes of drug-related problems based on PCNE classification V9.01

Causes = 2041	n, %
*C1—Drug selection*	
C1.1*—*Inappropriate drug according to guidelines/formulary	19 (0.9)
C1.2*—*No indication for drug	65 (3.2)
C1.3*—*Inappropriate combination of drugs, or drugs and herbal medications, or drug and dietary supplements	13 (0.6)
C1.5*—*No or incomplete drug treatment in spite of existing indication	32(1.6)
C1.6*—*Too many different drugs/active ingredients prescribed for indication	35 (1.7)
*C2-Drug form*	
C2.1*—*Inappropriate drug form/formulation (for this patient)	7 (0.3)
*C3.1—Dose selection*	
C3.1*—*Drug dose too low	563 (27.6)
C3.2*—*Drug dose of a single active ingredient too high	298 (14.6)
C3.3*—*Dosage regimen not frequent enough	218 (10.7)
C3.4*—*Dosage regimen too frequent	178 (8.7)
C3.5*—*Dose timing instructions wrong, unclear or missing	7 (0.3)
*C4—Treatment duration*	
C4.2*—*Duration of treatment too long	33 (1.6)
*C5—Dispensing*	
C5.1*—*Prescribed drug not available	1 (0.05)
*C6—Drug use process*	
C6.1*—*Inappropriate timing of administration or dosing intervals by a health professional	17 (0.8)
C6.2*—*Drug under- administered by a health professional	1 (0.05)
C6.3*—*Drug over- administered by a health professional	2 (0.1)
C6.4*—*Drug not administered at all by a health professional	7 (0.3)
C6.5*—*Wrong drug administered by a health professional	2 (0.1)
*C9—Other*	
C9.1*—*No or inappropriate outcome monitoring (incl. TDM)	2 (0.1)
C9.2*—*Other cause; specify^a^	541 (26.5)

^a^ Incomplete medication orders are one of the main causes, which fall in this subcategory.

2041 causes of DRPs, were identified. One problem can have more causes.

*TDM*, therapeutic drug monitoring.

### 3.5 Interventions and acceptance of the performed interventions

With an average of 1.3 interventions per DRP, 2,149 interventions were administered to manage 1,689 DRPs. Thirty-four problems received no intervention. The drug level (I3) domain accounted for 1,487 interventions (69.2%), while 367 interventions (17%) were conducted in the other intervention (I4) domain. Only 295 interventions (13.7%) were implemented at the prescriber level (I1) domain, such as ordering laboratory values, of which 95% were recommended to the prescriber.

The most prevalent subdomain among the interventions was dosage change (I3.2), which accounted for 950 instances (44.2%). Other interventions (specify) (I4.1) ranked second with 367 cases (17.1%), and instructions for use changed (I3.4), with 312 cases (14.5%). Of all the interventions, 2,125 were accepted (98.8%) by physicians, while only 22 were rejected. Among the accepted interventions, 2000 (93%) were accepted and fully implemented (Table 4).

**TABLE 4:** Interventions made by pharmacists and acceptance of intervention proposals classified based on to PCNE V9.1

Domains and subdomain		n, %
Interventions^a^ (*n* = 2,149, %)	*I1 - * *At Prescriber level: Intervention through the prescriber*	
	I1.1*—*Prescriber informed only	38 (1.8)
	I1.3*—*Intervention proposed to prescriber	251 (11.7)
	I1.4*—*Intervention discussed with prescriber	6 (0.3)
	*I3—* *At drug level: Intervention by pharmacist directly by changing drug or indicating change in drug use*	
	I3.1*—*Drug changed to	14 (0.7)
	I3.2*—*Dosage changed to	950 (44.2)
	I3.3*—*Formulation changed to	8 (0.4)
	I3.4*—*Instructions for use changed to	312 (14.5)
	I3.5*—*Drug paused or stopped	145 (6.7)
	I3.6*—*Drug started	58 (2.7)
	*I4—* *Other intervention: Interventions not covered in the other subdomains.*	
	I4.1*—*Other intervention (specify)^b^	367 (17.1)
Acceptance of Intervention proposals **^c^**	*A1—Intervention accepted by prescriber*	
	A1.1*—*Intervention accepted and fully implemented	2000 (93)
	A1.2*—*Intervention accepted, partially implemented	6 (0.3)
	A1.3*—*Intervention accepted, but not implemented	118 (5.5)
	A1.4*—*Intervention accepted, information unknown	1 (0.05)
	*A2—Intervention not accepted by prescriber*	
	A2.2*—*Intervention not accepted: no agreement	20 (0.9)
	A2.3*—*Intervention not accepted: other reason (specify)	2 (0.09)
	*A3—* *Other: No intervention proposed or acceptance unknown (no information)*	
	A3.1*—*Intervention not proposed	2 (0.09)

^a^ The term “intervention” describes the actions taken by a clinical pharmacist to address drug-related problems (DRPs). A single problem may require multiple interventions.

^b^ as many prescriptions were incomplete, the drugs’ doses were calculated by clinical pharmacists.

^c^ Each intervention suggestion will have one acceptance status.

### 3.6 Outcomes of interventions

Among the 1723 DRPs, 1,585 DRPs (92%) were resolved (O1.1), while 109 DRPs (6.3%) remained unresolved (O3).

Within the unresolved domain (O3), the lack of cooperation of the prescriber (O3.2) and the absence of a need or possibility to solve the problem (O3.4) were identified as the fundamental causes of the failed intervention outcomes (Table 5).

**TABLE 5:** Status of the drug-related problem classified based on PCNE V9.1

Status of the drug-related problem	n, %
***O0 - Not Known***	
** O**0.1*—* **Problem status unknown**	25 (1.5)
***O1 - Solved***	
** O1.1** *—* **Problem totally solved**	1,585 (92)
***O2 - Partially solved***	
** O2.1** *—* **Problem partially solved**	4 (0.2)
***O3*** *—* ***Not solved***	
** O3.2** *—* **Problem not solved, lack of cooperation of prescriber**	46 (2.7)
** O3.3** *—* **Problem not solved, intervention not effective**	9 (0.5)
** O3.4** *—* **No need or possibility to solve problem**	54 (3.1)

**The status of the drug-related problem reflects the outcome of the intervention. Only one level of problem solving can result from a single problem (or the combination of multiple interventions)**.

Out of the 46 unresolved DRPs due to lack of cooperation of prescribers (O3.2), 21 problems (45.7%) were classified as” unnecessary drug treatment problems (P3.1),” followed by 11 problems (23.9%) being classified as “adverse drug event (possibly) occurring (P2.1),” and seven problems (15.2%) were categorized as “effect of drug treatment not optimal (P1.2).”

Regarding the nine unresolved problems due to ineffective intervention outcomes (O3.3), six problems (66.7%) were classified as “unnecessary drug treatment problems (P3.1).”

Among the 54 unresolved problems due to the lack of need or possibility to solve the problem (O3.4), 19 problems (35.2%) were classified as “ADEs (possibly) occurring (P2.1),” followed by 15 problems (27.8%) being classified as “unnecessary drug treatment problems (P3.1).”

### 3.7 Significance of interventions

A French scale, adapted from Hatoum et al., was used to assess the significance of the 2,149 interventions (Table 6). Of these, 2,143 interventions (99.7%) were believed to have a significant impact on the patient safety, quality of life and treatment efficacy. Only six interventions (0.3%) were implemented after the event and displayed no significant impact. No interventions illustrated a very substantial or vital impact in this study.

**TABLE 6:** Significance of interventions according to the French scale adapted from Hatoum et al.

Description	Level	*n* = 2,149, (%)
***No impact on patient***	0	6(0.3)
**The intervention has an economic goal, or is done after the event, without consequences for the patient**		
***Significant impact***	I	2,143(99.7)
**The PI increases treatment’s efficacy or/and patient’s safety or/and his quality of life**		
***Very significant impact***	II	0(0)
**The PI avoids an organic dysfunction, an intensive care survey or a non-reversible sequel**		
***Vital impact***	III	0(0)
**The PI avoids a potentially fatal accident**		

***PI*, pharmacist intervention**.

An example of an intervention without impacting the patient is when the patient received a double dose of caffeine during the night shift when no pharmacist was present to detect the problem before reaching the patient. Conversely, an example of an intervention that significantly improved safety was when the pharmacist adjusted metronidazole due to the presence of liver disease by decreasing the dose by 50%.

### 3.8 Drugs involved in DRPs

A total of 1888 drugs were recorded, with 1726 involved in DRPs. These ten drugs (ampicillin-sulbactam, amikacin, meropenem, cefotaxime, piperacillin-tazobactam, metronidazole, cefepime, linezolid, caffeine, and dopamine) represented 75.3% of the drugs associated with problems and 69.2% of all prescribed drugs. The most frequently prescribed medications were ampicillin-sulbactam (288, 15.3%), amikacin (229, 12.1%), and caffeine (132, 7%) and both metronidazole and meropenem were each prescribed 116 times, accounting for 6.1% of all prescriptions. Ampicillin-sulbactam, amikacin, and meropenem were the most involved medications in DRPs (Table 7).

**TABLE 7:** The top ten medications involved in drug-related problems distributed by type of problem.

Medication	Number of times prescribed (1888)	Cases of DRP (1726)	Type of DRPs		
			Treatment effectiveness	Treatment safety	Other
**Ampicillin-sulbactam**	288 (15.3%)	255 (14.8%)	185 (10.7%)	44 (2.6%)	26 (1.5%)
**Amikacin**	229 (12.1%)	178 (10.3%)	88 (5.1%)	78 (4.5%)	12 (0.7%)
**Meropenem**	99 (5.2%)	161 (9.3%)	88 (5%)	22 (1.3%)	51 (3%)
**Cefotaxime**	116 (6.1%)	126 (7.3%)	73 (4.2%)	27 (1.6%)	26 (1.5%)
**Piperacillin-Tazobactam**	89 (4.7%)	122 (7%)	49 (2.8%)	25 (1.5%)	48 (2.7%)
**Metronidazole**	116 (6.1%)	120 (7%)	53 (3%)	13 (0.8%)	54 (3.1)
**Cefepime**	86 (4.6%)	115 (6.7%)	64 (3.7%)	18 (1%)	33 (2%)
**Linezolid**	78 (4.1%)	93 (5.4%)	32 (1.9%)	19 (1.1%)	42 (2.4%)
**Caffeine**	132 (7%)	71 (4.1%)	18 (1%)	29 (1.7%)	24 (1.4%)
**Dopamine**	73 (4%)	59 (3.4%)	8 (0.5%)	37 (2.1%)	14 (0.8%)

The most commonly prescribed group was anti-infective drugs (1,217, 64.5%), followed by cardiovascular drugs (208, 11%) and nervous system drugs (205, 10.9%).

The group primarily involved in DRPs was anti-infective drugs (1,309, 76%), followed by cardiovascular drugs (148, 8.6%) and nervous system drugs (126, 7.2%). The present study revealed that medications with high-risk ratios were less frequently utilized. Specifically, alprostadil and K salts exhibited the highest risk ratios (Table 8).

**TABLE 8:** Distribution of the drugs with the highest risk ratios.

Drug	DRP(n = 1726,%)	Times prescribed (n = 1888,%)	Risk ratio
**Alprostadil**	**8 (0.5)**	**3**	**2.7**
**K salts**	**5 (0.3)**	**2**	**2.5**
**trimebutine maleate**	**34 (2)**	**15**	**2.3**
**Hydrocortisone**	**2 (0.12)**	**1**	**2**
**Immunoglobulin**	**5 (0.3)**	**3**	**1.7**
**Heparin**	**5 (0.3)**	**3**	**1.7**
**Meropenem**	**161 (9.3)**	**99**	**1.6**
**Ceftazidime**	**27 (1.6)**	**17**	**1.6**
**midazolam**	**9 (0.5)**	**6**	**1.5**
**Piperacillin-Tazobactam**	**122 (7)**	**89**	**1.4**
**Enoxaparin**	**4 (0.23)**	**3**	**1.3**
**Cefepime**	**115 (6.7)**	**86**	**1.3**
**Epinephrine**	**5 (0.3)**	**4**	**1.3**

^a^ Risk ratio is calculated by dividing the number of DRP, by the total number of times the drug was prescribed.

### 3.9 Preventability of DRPs

Most identified DRPs were preventable, 98.7% (1701 cases) and only 1.3% (22 cases) of the DRPs were not avoidable.

**FIGURE 3:**
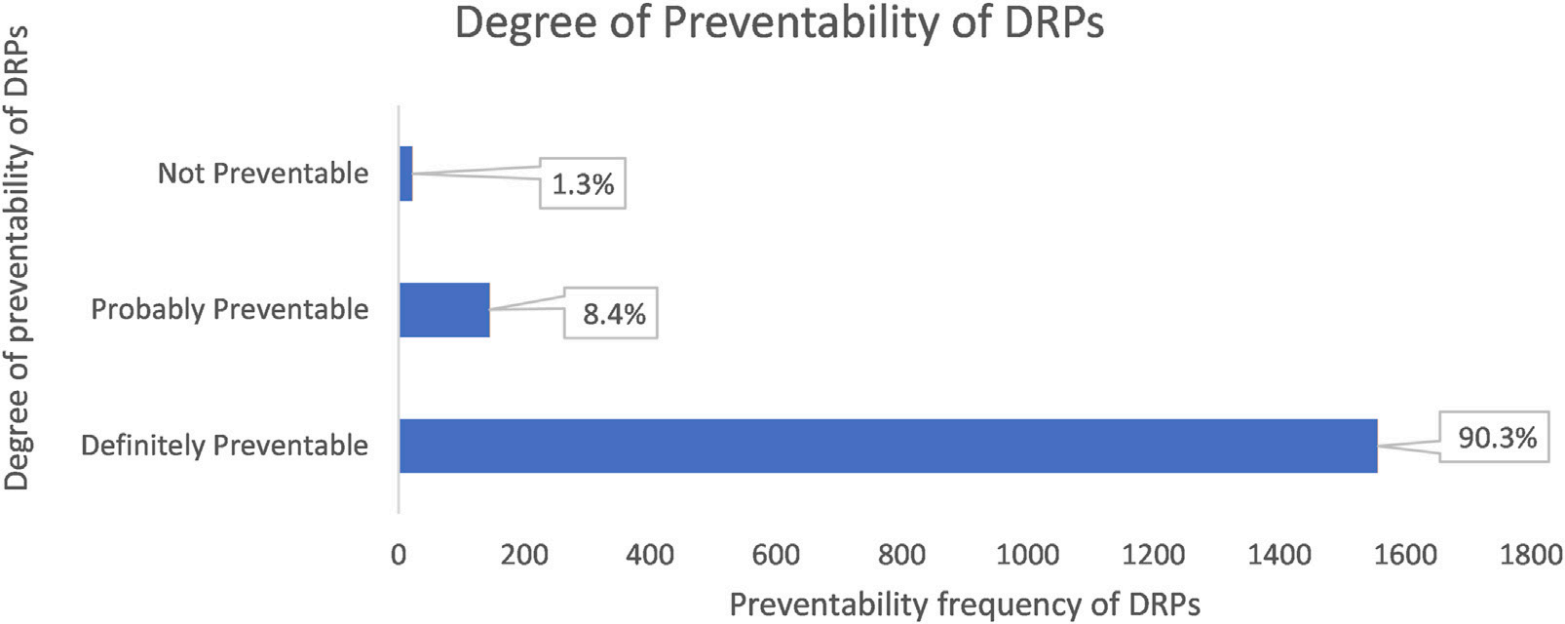
The chart shows the degree of preventability of DRPs classified according to the modified Schumock and Thorton’s scale.

81.1% (1,398 cases) of the preventable DRPs were related to dosage, frequency, and mode of administration; this was followed by drugs that were not appropriate for the patient’s condition (9.2%; 158 cases) and insufficient preventative measures (7%; 121 cases) (Figure 3).

### 3.10 Determinant factors associated with DRPs

Univariate binary logistic regression results revealed that DRPs were significantly associated with the number of medications, the number of clinical conditions, infectious disease presence and hospital stay length.

Compared to patients who used fewer than five medications, those who used five or more had a 12-fold increased risk of developing DRPs (COR = 11.938, 95% CI = 3.561–40.020).

Patients with three or more clinical conditions were 5.7 times more likely to experience DRPs than those with one condition (COR = 5.695, 95% CI = 1.660–19.545).

Similarly, patients with a hospital stay of ≥12 days had a 20.2-fold increased risk of DRPs compared to those with shorter stays (COR = 20.163, 95% CI = 4.734–85.878).

In the multivariate analysis, only hospital stay, and the number of medications remained significantly associated with DRPs **(** Table 9).

**TABLE 9:** Univariate and multivariate logistic regression results of risk factors associated with drug-related problems in the NICU of Assiut University Children’s Hospital.

Risk factors	COR (95%CI)	*p*-Value	AOR (95%CI)	*p*-Value
*Gender*				
**Male**	1	(Reference)		
**Female**	1.279 (0.612–2.671)	0.513		
**Age in days**	1.02 (0.955–1,089)	0.562		
**Birth weight in kg**	0.761 (0.5–1.157)	0.201		
**Lower gestational age in weeks (<37** ** ** **W)**	1.832 (0.887–3.785)	0.102		
**PROM**	2.854 (0.659–12.356)	1.61		
**Vaginal delivery**	0.778 (0.32–1.893)	0.58		
Number of medications				
**< 5**	1	(Reference)	1	(Reference)
**≥ 5**	11.938 (3.561–40.020)	<0.001	4.255 (1.088–16.649)	0.037
*Number of clinical conditions*				
**1**	1	(Reference)	1	(Reference)
**2**	6.748 (2.277–19.994)	0.001	2.639 (0.693–10.049)	0.155
**≥ 3**	5.695 (1.660–19.545)	0.006	1.644 (0.351–7.694)	0.528
*Disease category*				
**Malformations**	1.816 (0.790–4.172)	0.16		
**Respiratory disorders**	1.163 (0.551–2.458)	0.692		
**Infectious diseases**	4.145 (1.898–9.052)	<0.001	0.864 (0.297–2.509)	0.788
*Hospital stay*				
**< 12 days**	1	(Reference)	1	(Reference)
**≥ 12 days**	20.163 (4.734–85.878)	<0.001	8.871 (1.944–40.478)	0.005

***PROM*, premature rupture of membrane**; ***COR*, crude odd ratio; *AOR*, adjusted odd ratio**.

Accordingly, patients who stayed ≥12 days had a 9-fold increased risk of developing DRPs than those who stayed <12 days (AOR = 8.871, 95% CI = 1.944–40.478).

Additionally, compared to patients who used fewer than five medications, those who took five or more medications had a 4.2-fold increased risk of developing DRPs (AOR = 4.255, 95% CI = 1.088–16.649).

## 4 Discussion

This study serves as an exploration of DRPs within Egyptian NICUs and the role of clinical pharmacists in addressing them. It sheds light on an overlooked gap in the literature, as only a few studies have previously addressed DRPs in NICUs.

In this study, the incidence of DRPs was 89.6%, surpassing rates reported in other studies on the neonatal population. For instance, in two studies conducted in Brazil in the NICU of a teaching maternity hospital that serves a referral center for high-risk pregnancies using PCNE V6.2, the incidences were 59.8% (Leopoldino et al., 2019a) and 60.5%, respectively (Leopoldino et al., 2019b). A study conducted on septic neonates in Ethiopia using Cipolle’s method reported an incidence of 48.8% (Awoke et al., 2021). Another study conducted on cardiac neonates in Brazil using PCNE V6.2 had an incidence of 74.6% (Nascimento et al., 2020). This variation in incidence could be caused by variations in settings, including the training levels of healthcare workers, classification systems and protocols.

The present result exceeds those of studies on the child population in Hong Kong (25.7%) (Rashed et al., 2014), the United Kingdom and Saudi Arabia (45.2%) (Rashed et al., 2012), Iran (80.4%) (Jafarian et al., 2019) and Malaysia (52.9%) using the PCNE V8.02 classification system (Hon et al., 2020).

However, the current results are lower than those of a study performed in Egypt within the medical wards of cardiac children, where nearly every patient experienced at least one DRP, for which the incidence of DRPs was 100%. Using different methods of classification, the most frequent issue was drug-drug interaction (45.69%); all of these interactions needed only close monitoring, and that is logical due to the presence of polypharmacy, followed by unnecessary medication (31.95%) and underdosing represented only 21.09% (Sabry et al., 2014).

This might be due to a difference in population, as this study focused only on high-risk neonates.

According to PCNE V9.1, the most frequent DRP was treatment effectiveness at 46.4%, mainly the treatment effect not optimal at 41.4%, caused by dose selection primarily being too low at 27.6%. This result aligns with other studies conducted on cardiac neonates in Brazil, where treatment effectiveness was reported to be 49% (Nascimento et al., 2020). Similarly, a study conducted in two large teaching hospitals in the United Kingdom and Saudi Arabia highlighted dosing problems, primarily attributing 31.6% to drug doses being too low or dosing intervals being too long. Overall, dosing problems accounted for a reported occurrence of 54% (Rashed et al., 2012). In Ethiopia, dosing problems were reported at 42.5%, with predominantly low doses (34.9%) (Birarra et al., 2017). Similarly, in the NICU of Brazil, the effectiveness of treatment was mainly affected by suboptimal drug treatment (52.8%), with a recorded rate of 54.2% (Leopoldino et al., 2019a).

Problems related to treatment effectiveness are prevalent in the NICU because of the quick weight fluctuations and organ maturation of neonates, necessitating frequent dose adjustments (Wilbaux et al., 2016).

The study’s results differed from the multicenter study in France, Quebec, Switzerland, and Belgium that included PICUs (pediatric intensive care units) and pediatric cardiology units, where the main DRPs were inappropriate administration technique (29%), untreated indication (25%), and supratherapeutic dose (11%). (Prot-Labarthe et al., 2013).

The medication with the most significant problems with treatment effectiveness was ampicillin-sulbactam because dose calculations are based only on the ampicillin content based on the postnatal age (PNA) and weight, which are sometimes associated with calculation errors leading to subtherapeutic doses. This finding aligns with a study in Ethiopia, where ampicillin was the most involved drug in dosing problems (Birarra et al., 2017). This can be explained by the fact that ampicillin is combined with aminoglycosides to treat suspected early-onset neonatal sepsis (Bradley, J.S., 2022).

Furthermore, amikacin is one of the most challenging drugs for which dosing schedules are optimized, as the dose regimen is based on the PNA and gestational age (GA), and the clearance of the drug is based on glomerular filtration, which is immature in neonates. Kidney functions rapidly change as neonates undergo progressive maturation, necessitating frequent dose adjustments (Wilbaux et al., 2016), which explains why amikacin is highly involved in DRPs. This finding is similar to what was observed in studies conducted in Hong Kong (Rashed et al., 2014) and Brazil (Leopoldino et al., 2019a), where gentamicin was the most implicated drug in DRPs in both studies. Given that all aminoglycosides have a large volume of distribution in neonates and that the progressive maturation of kidney functions leads to a decreased concentration of drugs, frequent dose adjustments are needed (Wilbaux et al., 2016).

The second most frequent DRP domain observed was the other domain, recorded at 27.2% of cases, mainly the subdomain unclear problem (20.2%). Since the physicians were responsible for calculating the dosages, incomplete prescriptions were the primary source of this issue.

The present result is different from the results of the Malaysian study. In Malaysia, the most frequent problem was treatment safety at 34.3%, higher than in this study, followed by “other domain” at 42.4%, mainly incomplete prescriptions (28.3%). The last was treatment effectiveness, recorded at 23.2%, primarily because of drug treatment not being optimal (17.7%) (Hon et al., 2020). However, there are noteworthy similarities in certain domains. In our study, the “Other” domain, encompassing issues beyond treatment safety and effectiveness, aligns with findings from Malaysia. Specifically, we observed a substantial proportion of cases categorized under the “Other” domain, accounting for 469 cases (27.2%). Within this category, the predominant issue was incomplete prescriptions (P3.2), contributing to 348 cases (20.2%). This finding mirrors the Malaysian study’s observation of incomplete prescriptions being a significant concern within the “Other” domain, which was reported at 28.3%. By highlighting these similarities, we aim to underscore the consistency of certain DRP categories across different healthcare settings, despite variations in overall prevalence rates.

The third most frequent DRP was treatment safety at 26.4%, which is similar to what was reported in the NICU of Brazil at 41.4% (Leopoldino et al., 2019a) and 54.4% (Nascimento et al., 2020), respectively. The present result was lower than these previous results.

The current study’s findings contrast those in Iranian and Ethiopian studies. The most frequent issue in these studies was treatment safety at 43.5%, surpassing the incidence observed in the current study. This was followed by treatment efficacy, mainly the effect of therapy not being optimal (22%), which was reported at 36.8% (Jafarian et al., 2019). In addition, a dose too high was the most common problem recorded at 34.7%, followed by a dose too low at 19.8% (Awoke et al., 2021).

In this study, the most frequent cause of these problems was dose selection, followed by other causes. This result aligns with those of Iranian (Jafarian et al., 2019) and Brazilian studies (Leopoldino et al., 2019a).

Pediatric dosage errors can lead to toxicity from an overdose or ineffective treatment because of subtherapeutic concentrations. Weight-based dosing calculations increase the likelihood of incorrect doses in pediatrics compared to adults. DRPs are primarily caused by performance deficits, knowledge deficits, staff shortages, and heavy workloads for junior doctors and nurses (Lesar et al., 1997).

Clinical pharmacists conducted a total of 2,149 interventions. These interventions relied on the types of defined DRPs, where dosage change was the most frequent intervention, followed by other interventions and providing instructions for use. These findings are consistent with studies conducted in Hong Kong (Rashed et al., 2014) and in the United Kingdom and Saudi Arabia (Rashed et al., 2012). The acceptance rate was notably high at 98.8%, with 93% of these interventions being accepted and fully implemented. This result is comparable to the results reported in other studies in pediatric wards in four French-speaking countries (97.9%) (Prot-Labarthe et al., 2013), in pediatric patients with kidney disease (99.5%) (Ibrahim et al., 2013) and >90% in both the NICU of Brazil and in cardiac neonates in the NICU (Leopoldino et al., 2019a; Nascimento et al., 2020). These results are higher than those detected in Malaysia (>80%) (Hon et al., 2020), in cardiac pediatric patients in Egypt (65%) (Sabry et al., 2014), and in Iran (59.2%) (Jafarian et al., 2019).

The high acceptance rate reflects the openness of physicians to pharmacist interventions to optimize the healthcare services provided to patients.

As a result, 92.2% (1,589) of the DRPs were either fully or partially resolved; only 109 problems remained unresolved. The main reasons for unresolved problems included situations where the patient died or was discharged before any action could be taken as well as a lack of cooperation from the prescriber. This is comparable to another study on children with renal diseases, in which 96% of DRPs were resolved (Ibrahim et al., 2013).

This finding highlights the importance of clinical pharmacy activities. Clinical pharmacists performed while participating in ward rounds and provided reasonable recommendations to healthcare workers, including physicians and nurses, to identify, prevent, and resolve DRPs, ultimately leading to improved patient outcomes.

Of these interventions, 99.7% may have had a significant impact on patient safety and treatment efficacy, while only 0.3% having no impact as the intervention occurred after the event. This result is comparable to a study conducted by (Strong and Tsang, 1993) which showed that 93% of interventions had a positive impact on patient outcomes. Additionally, it surpasses the percentages reported by (Fernández-Llamazares et al., 2012) (78.6%), using a slightly modified version of the scale designed by Overhage et al., and by (Virani and Crown, 2003) (86%).

The high mortality rate observed in this study can be attributed to the complexity of the patient’s conditions. Deceased patients were detected to have congenital anomalies and sepsis, which are prevalent conditions within the unit, highlighting the significance of infectious diseases and congenital anomalies as primary contributors to mortality.

In the present study, most identified DRPs were preventable (98.7%). This result aligns with similar studies; for example, in the NICU of Brazil, 90% of DRPs were preventable (Leopoldino et al., 2019a); in Hong Kong, 81.7% of DRPs were preventable (Rashed et al., 2014); and 80.3% of DRPs were preventable in both studies that were conducted on hospitalized children in the United Kingdom and Saudi Arabia and tertiary critical care pediatric settings in Saudi Arabia (Rashed et al., 2012; Tawhari et al., 2022). Additionally, in another study in pediatric units in Saudi Arabia, 87.7% of DRPs were preventable (Alazmi et al., 2019).

To the best of the authors’ knowledge, the tool used to evaluate the preventability of DRPs has been used previously employed in previous studies (Rashed et al., 2012; 2014) and was developed to assess adverse drug reactions.

NICU clinical pharmacy services should be strengthened as one means of preventing DRPs. More training programs on neonate pharmacology and pharmacotherapy should be developed for medical professionals, including physicians and nurses.

The most involved groups were anti-infective, cardiovascular, and nervous system drugs. This finding is similar to what was discovered in other studies: anti-infective drugs were the most involved group in DRPs in Hong Kong (Rashed et al., 2014), the United Kingdom and Saudi Arabia (Rashed et al., 2012), Brazil (Leopoldino et al., 2019a; 2019b; Nascimento et al., 2020), and Ethiopia (Birarra et al., 2017; Awoke et al., 2021).

Anti-infective drugs were frequently prescribed due to the high prevalence of infectious diseases, and among patients, particularly neonates, who are more susceptible to infections owing to low immunity and prematurity.

Blix et al. were the first to describe the risk ratio (Blix et al., 2004). In the present study, this method was used to identify the risk ratio associated with prescribed medications. Interestingly, the present investigation revealed that medicines with the highest risk ratio were the least prescribed. This result is similar to what was reported in the Blix study (Blix et al., 2004) and Brazil studies (Leopoldino et al., 2019b; Nascimento et al., 2020), which contradicted previous findings that claimed that a drug’s risk of adverse events increased with the number of prescriptions (Stavroudis et al., 2008; Pawłowska et al., 2016). This risk depends on the patient’s characteristics and the drug’s chemical and pharmacological properties (Nascimento et al., 2020). The risk ratio was highest for alprostadil, as this medication’s calculation is complex and needs special requirements for infusion to avoid adverse events.

Of the risk factors analyzed, only the number of medications, length of hospital stay, presence of infectious disease, and number of clinical conditions were significantly associated with DRPs, according to the univariate analysis, which aimed to identify risk factors related to DRPs. Univariate analysis revealed that gender, age, birth weight, mode of delivery, other disease categories, and lower gestational age were not significantly associated with DRPs. Subsequent multivariate analysis confirmed that only the number of medications and length of hospital stay remained significantly associated with DRPs. This result is consistent with previous studies conducted in Ethiopia (Dedefo et al., 2016) and Brazil (Leopoldino et al., 2019b). Other studies reported that polypharmacy was significantly associated with DRP (Rashed et al., 2012; 2014; Ibrahim et al., 2015; Birarra et al., 2017; Awoke et al., 2021). The risk of patients having DRPs increases with the complexity of their medication regimen.

### 4.1 Strengths and limitations

This study possesses its own strengths and limitations. Among its strengths, it is the first study to describe the incidence of DRPs, their characteristics, and the impact of clinical pharmacy interventions in Egypt’s NICU. Additionally, the study covered all aspects of DRPs using a widely accepted DRP classification system. Moreover, this was a prospective study in which intense follow-up was allowed, which helped identify additional DRPs.

However, the study’s limitations include that it was conducted in a single NICU, which may affect the generalizability of the results.

### 4.2 Future recommendations

To enhance the generalizability of findings, conducting a multicenter study is recommended.

Further research is required to determine the cost savings associated with clinical pharmacist interventions. Additionally, the incidence and types of adverse drug reactions were not investigated in this study and need further investigation.

Moreover, additional research is warranted to investigate the pharmacist’s role in preparing and optimizing the use of electrolyte solutions and total parenteral nutrition (TPN).

## 5 Conclusion

In conclusion, this study revealed a high prevalence of DRPs in the Egyptian NICU. The most predominant problem was treatment effectiveness, mainly the effect of treatment not being optimal due to dose selection. The pharmacists’ interventions were highly accepted, with an acceptability level of 98.8%, resulting in the resolution of 92% of the identified DRPs. Anti-infective drugs were the most involved group in the DRPs. Polypharmacy and length of hospital stay emerged as significant risk factors for DRPs. The presence of a clinical pharmacist in the unit helped identify, prevent, and resolve DRPs, enhancing patient safety, treatment efficacy, and quality of life.

## Data Availability

The raw data supporting the conclusion of this article will be made available by the authors, without undue reservation.
